# *SPSL1* is essential for spermatophore formation and sperm activation in *Spodoptera frugiperda*

**DOI:** 10.1371/journal.pgen.1011073

**Published:** 2023-12-04

**Authors:** Lansa Qian, Xu Yang, Xiaomiao Xu, Dehong Yang, Chenxu Zhu, Meiyan Yi, Honglun Bi, Yaohui Wang, Yongping Huang

**Affiliations:** 1 Key Laboratory of Insect Developmental and Evolutionary Biology, Center for Excellence in Molecular Plant Sciences, Shanghai Institute of Plant Physiology and Ecology, Chinese Academy of Sciences, Shanghai, China; 2 University of Chinese Academy of Sciences, Beijing, China; 3 Department of Pharmacology and Cancer Biology, Duke University School of Medicine, Durham, North Carolina, United States of America; 4 Anhui Province Key Laboratory of Crop Integrated Pest Management, College of Plant Protection, Anhui Agricultural University, Hefei, China; 5 State Key Laboratory of Microbial Metabolism/School of Environmental Science and Engineering, Shanghai Jiao Tong University, Shanghai, China; 6 State Key Laboratory of Cotton Biology, School of Life Sciences, College of Agriculture, Henan University, Kaifeng, China; University of Kentucky, UNITED STATES

## Abstract

The reproductive process in various species has undergone evolutionary adaptations at both the physiological and molecular levels, playing a significant role in maintaining their populations. In lepidopteran insects, the spermatophore is a unique structure formed in the female reproductive system, in which sperm storage and activation take place. It is known that the formation of the spermatophore is regulated by seminal fluid proteins derived from males. However, studies investigating the genetic mechanisms behind spermatophore formation in lepidopterans have been limited. In this study, our focus was on *SPSL1*, a gene that encodes a trypsin-type seminal fluid protein in *Spodoptera frugiperda*, a pest species with global invasive tendencies. Our findings revealed that *SPSL1* expression was predominantly observed in the male reproductive tracts, and the disruption of this gene resulted in male sterility. Surprisingly, fluorescence analysis indicated that the absence of *SPSL1* did not affect spermatogenesis or sperm migration within the male reproductive system. However, when females mated with *SPSL1*-mutant males, several defects were observed. These included disruptions in spermatophore formation, sperm activation in the copulatory bursae, and sperm migration into the spermathecae. Additionally, mass spectrometry analysis highlighted reduced levels of energy-related metabolites, suggesting that *SPSL1* plays an essential role in promoting hydrolysis reactions during copulation. Consequently, our study demonstrates that *SPSL1* is crucial for male fertility due to its functions in spermatophore formation and sperm activation. This research provides valuable insights into the genetic factors underlying reproductive processes in lepidopteran insects and sheds light on potential strategies for controlling invasive pest populations.

## Author summary

In lepidopteran insects, males produce a spermatophore to deliver the sperm during copulation and promote reproductive success. Seminal fluid plays a crucial role in the formation of spermatophore and the regulation of sperm activation. However, little is known about the semen protein molecules involved in these processes. In this work, we used *Spodoptera frugiperda* as a model to study the function of the seminal fluid protein SPSL1, a trypsin-type serine protease, in the reproductive process. By analyses of insects with a loss-of-function mutation in *SPSL1*, we found that SPSL1 affects the structure of the spermatophore, sperm activation, sperm distribution, and sperm migration in the female reproductive tract. Our research deepens our understanding of the reproductive process of lepidopterans and demonstrates that *SPSL1* is a potential target for novel pest control methods such as sterile insect technology.

## Introduction

The successful reproduction of animals relies on various physiological and behavioral processes that occur in a coordinated manner between mating partners [1]. The insects, for instance, transfer seminal fluid proteins (SFPs) from males to females during mating, which serve as both structural components and regulators of reproductive behavior in females [2]. In *Drosophila melanogaster*, several SFPs have been identified and characterized for their roles in regulating sexual receptivity, oviposition, sperm storage, sperm competition, and mating plug formation in females, including Acp70A/sex peptide, Dup99B, Acp26Aa, Acp36DE, PEBme [3–7]. However, it is important to note that there are significant differences in genetic and molecular mechanisms controlling sexual reproduction among different insect species [8]. While much of the research on SFPs has focused on *Drosophila*, it is crucial to acquire knowledge about the evolutionary diversity of SFPs in other insect groups.

Lepidoptera is an emerging and noteworthy taxon for reproduction research [9–11]. Understanding the reproductive process in this group is valuable due to the inclusion of both pest species and economically important species [12, 13]. There are substantial differences in reproduction between lepidopteran and dipteran insects, such as sex determination mechanisms, spermatogenesis, and fertilization processes [14–16]. Notably, lepidopteran males exhibit dichotomous spermatogenesis, producing eupyrene sperm (with nuclei) and apyrene sperm (without nuclei) within a single individual male [15]. Both types of sperm are essential for fertilization. Eupyrene sperm carries DNA and fertilizes eggs, while apyrene sperm aids in the transportation of eupyrene sperm to female sperm-storage organs [17–19]. In the reproductive system of female lepidopterans, the spermatophore is a specialized structure formed during mating that serves as a sperm-delivery device. Within the spermatophore, eupyrene sperm bundles dissociate, apyrene sperm gain motility, and are ultimately transferred into the spermatheca for egg fertilization [20, 21]. It has been reported that both types of sperm and SFPs are ejaculated by males and transferred to females, which are crucial for spermatophore formation during copulation [22, 23]. Despite extensive characterization of SFPs in lepidopterans, the understanding of specific factors involved in reproduction remains limited, particularly in regard to the molecular mechanisms underlying processes such as sperm activation and spermatophore formation.

SFPs in lepidopteran insects, including *Bombyx mori*, *Spodoptera litura*, *Plutella xylostella*, have been found to contain numerous trypsin-type serine proteases and play important roles in male reproduction [24–26]. In *B*. *mori*, a trypsin-type protease called Initiatorin, also known as Serine Protease 2 (Ser2), has been identified as a sperm activation factor through in vitro enzyme treatment experiments [27]. Recent studies on *B*. *mori* have demonstrated that knockout of *Ser2* or *Serine Protease 1* (*Ser1*) leads to male sterility [28, 29]. While some studies have explored the reproductive effects of trypsin-type serine proteases in lepidopteran insects, their specific roles in regulating processes like spermatogenesis, sperm activation, and spermatophore formation remain unclear.

The fall armyworm, *Spodoptera frugiperda*, is a globally widespread invasive pest that causes significant damage to economically important crops [30]. However, the molecular mechanisms involved in reproduction of *S*. *frugiperda* are poorly understood. In this study, we identified and characterized the physiological function of the *Serine Protease Snake-like 1* (*SPSL1*) gene in *S*. *frugiperda*. We observed that *SPSL1* is predominantly expressed in the internal reproductive organs of males, and loss-of-function mutants of *SPSL1* exhibited significantly reduced fertility in males. Fluorescence staining assays revealed that dichotomous spermatogenesis was unaffected in *SPSL1*-mutant males. However, females mated with *SPSL1*-mutant males experienced disruptions in spermatophore formation, sperm activation, and sperm migration. Furthermore, mass spectrometry analysis indicated a reduction in energy-related metabolites in the copulatory bursae of females mated with *SPSL1*-mutant males. Overall, our study contributes to the understanding of specific SFP functions and provides a potential gene target for pest control.

## Results

### *SPSL1* is predominantly expressed in male internal genitalia

**Fig 1:**
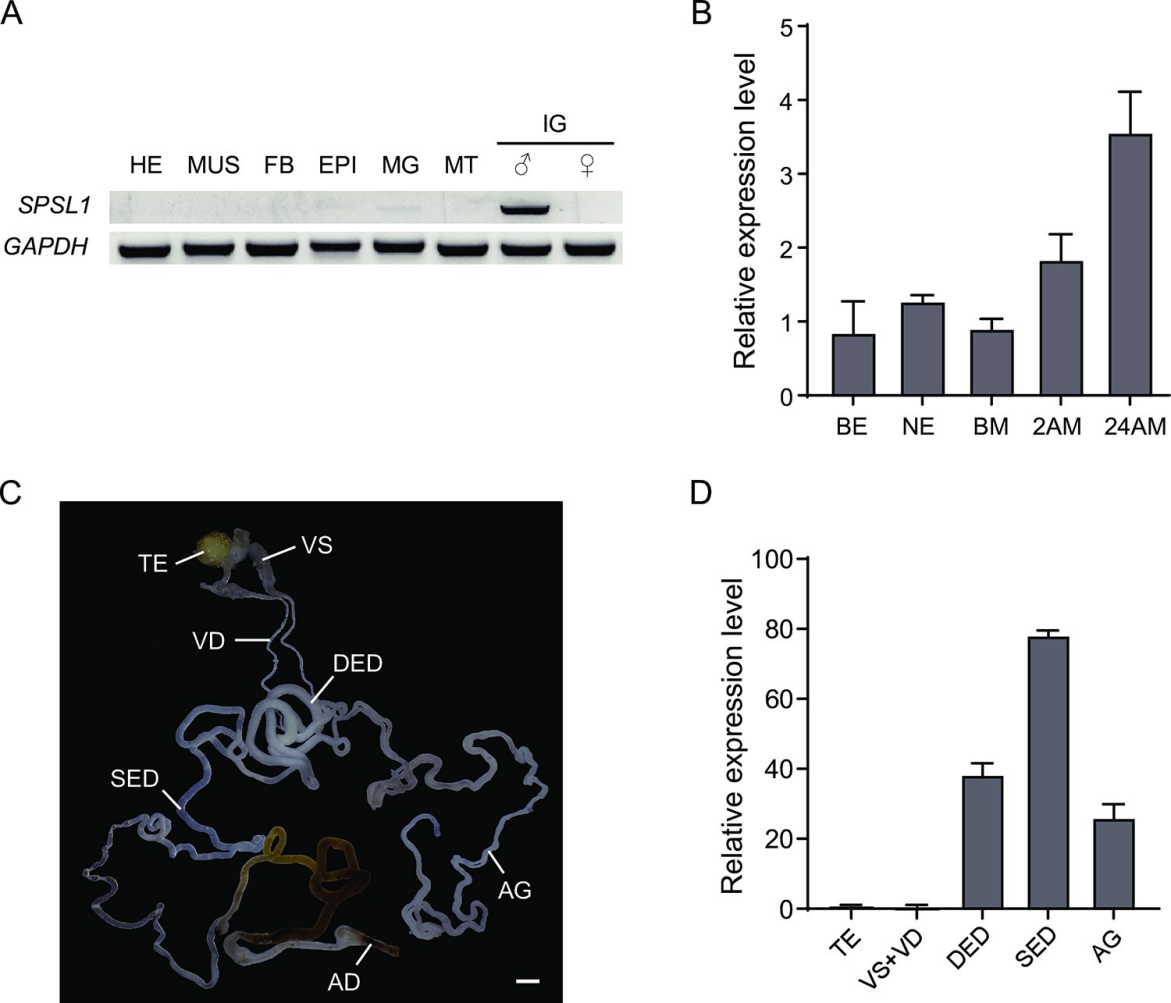
*SPSL1* is predominantly expressed in male internal genitalia. (A) Semi-quantitative RT-PCR analysis of *SPSL1* mRNA levels in indicated adult tissues (n = 10). Abbreviations: HE, head; MUS, muscle; FB, fat body; EPI, epidermis; MG, midgut; MT, Malpighian tubules; IG, internal genitalia. The samples other than IG are from the same number of male and female adults. (B) qRT-PCR analysis of *SPSL1* mRNA levels in male internal genitalia at indicated times. BE, Before eclosion; NE, New eclosion; BM, Before mating; 2AM, 2h after mating; 24AM, 24h after mating. (C) Schematic of the male reproduction system of *S*. *frugiperda*. Abbreviations: TE, testis; VS, vesiculae seminales; VD, vas deferens; DED, double ejaculation ductus; SED, single ejaculation ductus; AG, accessory glands; AD, adeodatus. Scale bar, 1mm. (D) qRT-PCR analysis of *SPSL1* mRNA levels in indicated male internal genital tissues. *SfGADPH* was used as an internal reference. Data are means ± SEM.

Our previous research found that mutation of *Ser1* leads to male sterility in *B*. *mori* [28], however, how *Ser1* is particularly involved in the reproduction process remains unknown. We subsequently identified the gene in the *S*. *frugiperda* Genbank RefSeq database annotated as “*Serine Protease Snake-like*” (accession number: XP_035457022.2) and we propose to rename this gene as *SPSL1* (for *Serine Protease Snake-like 1*). Phylogenetic analysis of a total of 19 trypsin-type serine proteases resulted in five distinct clades and revealed that the SPSL1 protein of *S*. *frugiperda* is clustered with the Lepidoptera clade (S1A Fig). The amino acid sequences of SPSL1 are highly conserved among seven species of Lepidoptera including *S*. *frugiperda*, *S*. *litura*, *Trichoplusia ni*, *Helicoverpa zea*, *Maniola jurtina*, *Manduca sexta*, and *B*. *mori* (S1B Fig). We then performed semi-quantitative RT-PCR analysis of seven different tissues of *S*. *frugiperda* adults and found that *SPSL1* was predominantly expressed in the internal genitalia of male moths (Fig 1A). We then used qRT-PCR analysis to further detect the expression pattern of *SPSL1* during mating and observed that it gradually increased from 1 day before eclosion to 1 day after mating (Fig 1B). Within the male internal genitalia, *SPSL1* was highly expressed in single ejaculation ductus, double ejaculation ductus, and accessory glands (Fig 1C and 1D), implying that SPSL1 may have a role in male fertility.

### *SPSL1* mutation impairs male fertility

**Fig 2:**
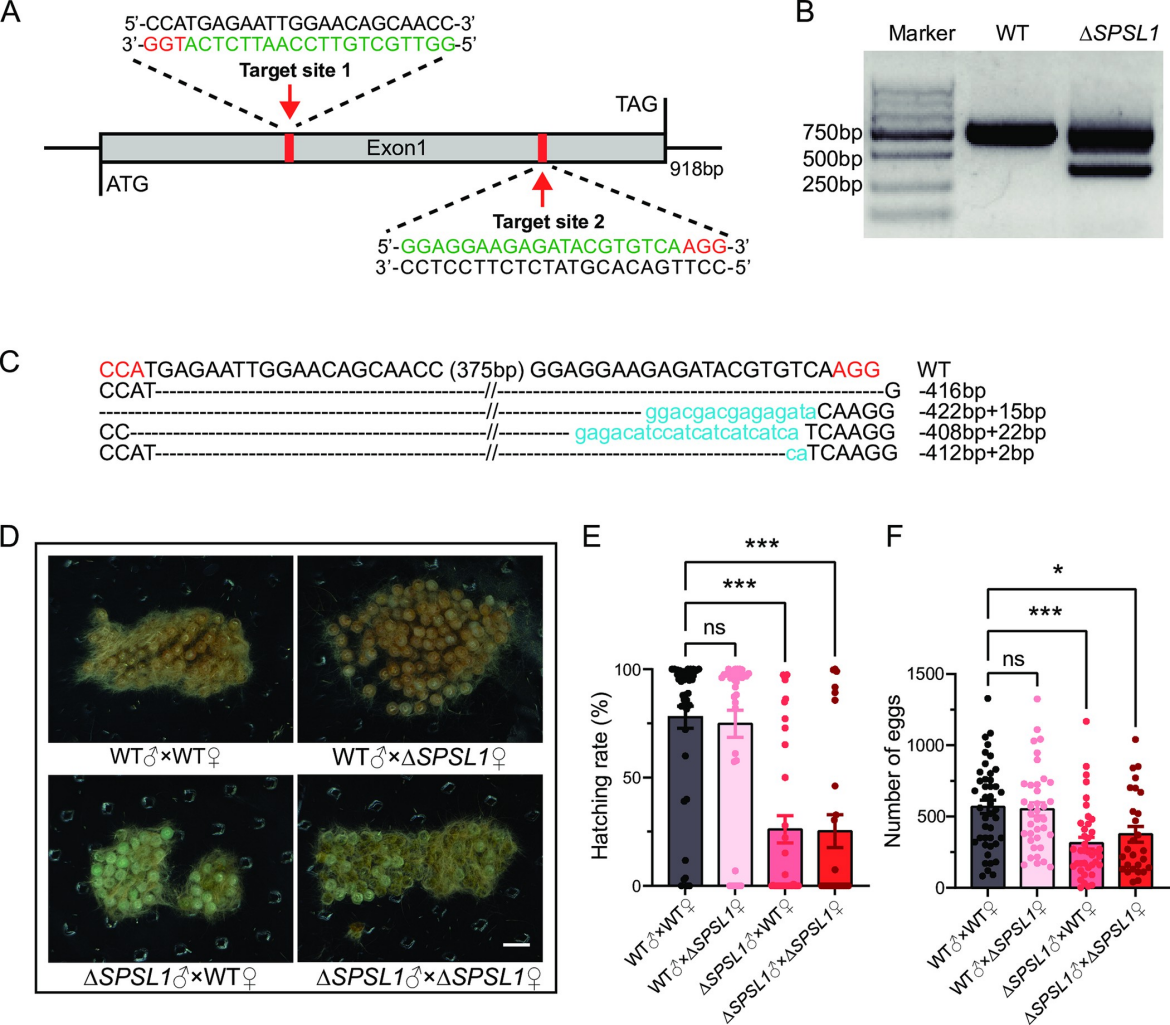
Mutation of *SPSL1* decreases male fertility. (A) Schematic of *SPSL1* gene structure and sgRNA target sites. The gray box indicates the exon. Red arrows indicate the target sites of sgRNA1 and sgRNA2. The target sequence and PAM sequence are highlighted in green and red, respectively. (B) Genomic PCR analysis of *SPSL1* mutation at embryo stage. A fragment of around 800 bp was detected in WT. Fragments around 800 bp and 400 bp were detected in *SPSL1* mutants. (C) The sequence of the region between sgRNA target sites in the *SPSL1* gene for WT and four randomly selected *SPSL1*-mutant G0 individuals. Dashed lines represent the deleted bases and the lowercase characters in blue represent the inserted bases. The net change in length is given to the right of each sequence (-, deletion; +, insertion). (D) Photographs of eggs produced by different crosses. Eggs were photographed 72 h after spawning. Scale bar, 1mm. (E) The hatching rate for indicated crosses (n > 26. *, *p* < 0.05; ***, *p* < 0.001. One-way ANOVA test). (F) The number of eggs laid for indicated crosses (n > 26. *, *p* < 0.05; ***, *p* < 0.001. Kruskal-Wallis test). Δ*SPSL1* represents *SPSL1* mutants.

To explore the biological function of *SPSL1*, we used the CRISPR/Cas9 system to engineer loss-of-function mutants. We designed two small guide RNAs (sgRNAs) targeting the only exon of *SPSL1* (Fig 2A). We confirmed that gene editing was successful by analysis of DNA extracted from approximately 30 randomly selected injected embryos and observed a shorter fragment in injected versus control embryos that is consistent with the expected size of the mutant gene region (Fig 2B). We then amplified and sequenced the *SPSL1* gene in four randomly selected representative G0 offspring. This confirmed that large deletions or insertions occurred in *SPSL1*-mutant individuals (Fig 2C). These results demonstrated that we successfully obtained the loss-of-function mutants of *SPSL1*. The *SPSL1* mutants were viable and grossly normal during all development stages. We then investigated whether the disruption of *SPSL1* affects the fertility of *S*. *frugiperda*. To do this, we performed fecundity assays. We found that the fertility of the *SPSL1*-mutant males was dramatically reduced compared to that of the wild-type (WT) males (Fig 2D–2F). The hatching rates of eggs laid by WT females and by *SPSL1*-mutant females mated with *SPSL1*-mutant males were only 26.10% and 25.26%, respectively. In contrast, when mated with WT males, 77.87% and 74.85% of eggs laid by WT females and by *SPSL1*-mutant females were hatched, respectively (Fig 2E). In addition, mating with *SPSL1*-mutant males significantly reduced the numbers of eggs laid (Fig 2F). When mated with *SPSL1*-mutant males, WT females and *SPSL1*-mutant females laid on average 312 and 375 eggs, respectively; when mated with WT males, the numbers were 567 and 551 eggs on average per female, respectively. Taken together, these results demonstrate that *SPSL1* is essential for male fertility.

### *SPSL1* is not required for dichotomous spermatogenesis or sperm migration in males

The demonstration that *SPSL1*-mutant males are sterile indicates that there may be defects in the male reproductive system. Therefore, we first investigated whether the *SPSL1*-mutant males have gross defects in the genitalia or the reproductive system. However, no obvious defects were detected (S2A–S2B’ Fig), so we searched for anomalies in spermatogenesis. In lepidopteran insects investigated, males have two morphs of sperm, and both are essential for fertility [17–19]. To date, there have been no reports describing the morphology of sperm or the molecular mechanism of spermatogenesis in *S*. *frugiperda*. Therefore, to evaluate the sperm morphology and to determine whether *SPSL1* is involved in spermatogenesis in *S*. *frugiperda*, we performed fluorescence staining of sperm released from the testes on the second day after eclosion.

**Fig 3:**
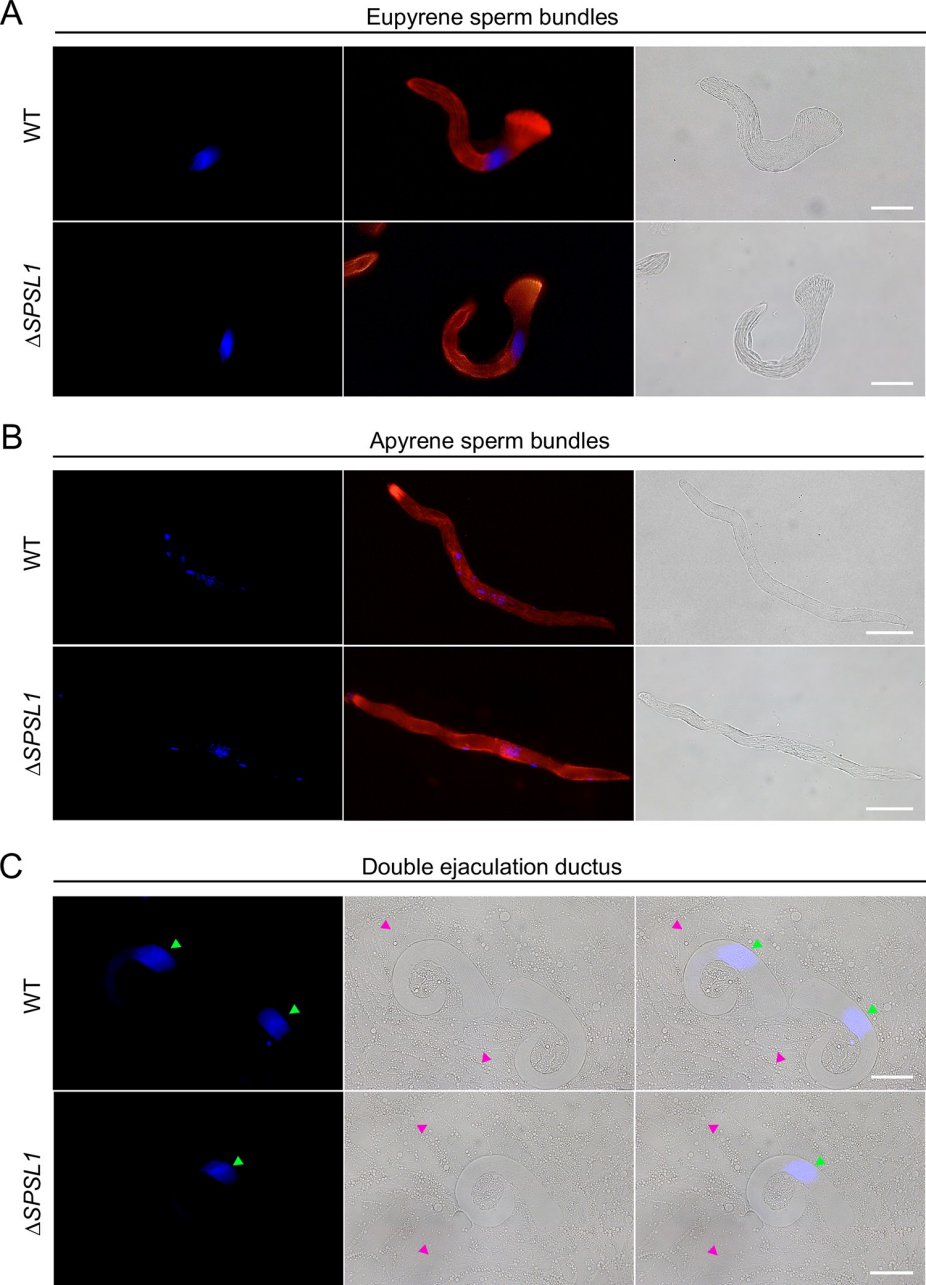
*SPSL1* mutation does not affect spermatogenesis or sperm migration in the male reproduction system. (A) Representative immunofluorescence images of eupyrene sperm bundles released from the testes of WT and *SPSL1*-mutant males on the second day after eclosion. (B) Representative immunofluorescence images of apyrene sperm bundles released from the testes of WT and *SPSL1*-mutant males on the second day after eclosion. (C) Representative immunofluorescence images of eupyrene sperm bundles (green arrow) and apyrene sperm (magenta arrow) released from the double ejaculation ductus of WT and *SPSL1*-mutant males. Scale bars, 50μm. Blue, Hoechst; Red, F-actin. Δ*SPSL1* represents *SPSL1* mutants.

We observed two morphs of sperm bundles: One type was thick and had a ‘brush-like’ head, and nuclei were neatly arranged near the head (Fig 3A); The other type of sperm bundle was slender, and had irregularly distributed nuclei near the center of the bundle (Fig 3B). We further distinguished the eupyrene sperm bundles and apyrene sperm bundles by using nuclei fluorescence staining of sperm released from double ejaculation ductus, in which apyrene sperm bundles were typically dissociated and nuclei released. We did not observe the slender sperm bundles while only ‘brush-like’ head sperm bundles with neatly arranged nuclei were detected (Fig 3C). This result indicated the ‘brush-like’ head sperm bundles were eupyrene while the others were apyrene. However, we did not observe any obvious differences between WT and *SPSL1* mutants in morphs of sperm bundles released from the testes or double ejaculation ductus (Fig 3A–3C), suggesting that the disruption of *SPSL1* did not affect spermatogenesis or sperm migration in males.

### *SPSL1* mutation leads to defects in spermatophore formation and sperm activation

**Fig 4:**
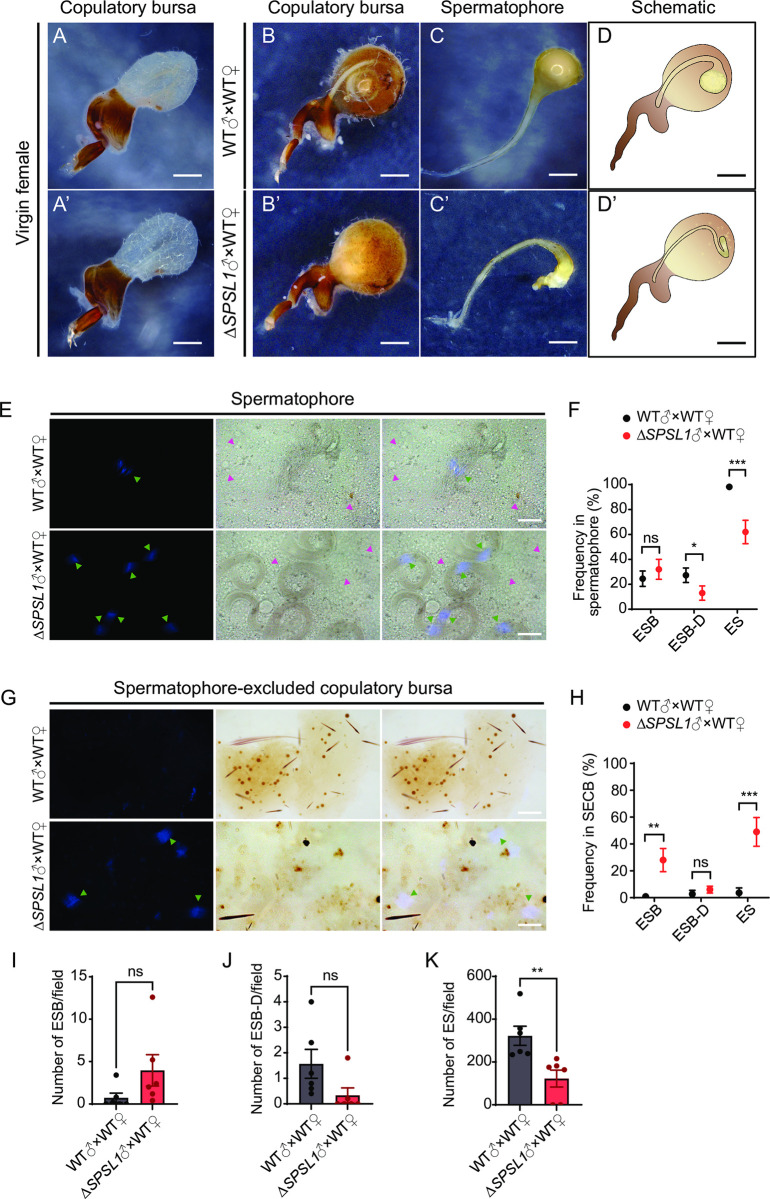
Mutation of *SPSL1* leads to defects in spermatophore formation, sperm distribution, and sperm activation. (A, A’) Representative images of the copulatory bursa of virgin WT female. Scale bars, 1mm. (B, B’) Representative images of copulatory bursa of WT females mated with WT or *SPSL1*-mutant males 1 hour-post copulation (hpc). Scale bars, 1mm. (C, C’) Representative images of spermatophore dissected from the copulatory bursa of females mated WT or *SPSL1*-mutant males 1 hpc. Scale bars, 1 mm. (D, D’) Schematic of the spermatophore inside in the copulatory bursa of WT females mated with WT or *SPSL1*-mutant males. scale bars, 1mm. (E) Representative images of sperm bundles released from spermatophores from indicated mating at 1 hpc. The green arrows indicate eupyrene sperm bundles, magenta arrows indicate apyrene sperm. Blue, Hoechst. Scale bars, 50 μm. (F) The frequencies of eupyrene sperm bundles (ESB), eupyrene sperm bundles in dissociation (ESB-D), eupyrene sperm (ES) released from spermatophores. (G) Representative images of sperm bundles released from spermatophore-excluded copulatory bursae at 1 hpc for indicated matings. The green arrows indicate eupyrene sperm bundles. Blue, Hoechst. Scale bars, 50 μm. (H) The frequencies of ESB, ESB-D, ES released from spermatophore-excluded copulatory bursae. (I-K) The numbers of I) ESB, J) ESB-D, K) ES per field of view released from copulatory bursae at 1 hpc for indicated matings. For F and H, 110 fields of view from 22 females mated with WT males and 100 fields of view from 20 females mated with *SPSL1*-mutant males were analyzed. For I-K, 30 fields of view from 6 females for indicated matings were analyzed. Data are means ± SEM (*p < 0.05; **p < 0.01; ***p < 0.001. two-tailed Student’s *t*-test). Δ*SPSL1* represents *SPSL1* mutants.

In lepidopteran females, the spermatophore consists of proteins from the seminal fluid that are transferred from males during copulation [23]. As the *SPSL1* mutation does not affect the male reproductive system, we then explored whether the *SPSL1* mutation influences spermatophore formation. For this purpose, we evaluated the copulatory bursa before and after mating, and the spermatophore 1 h post copulation (hpc) in WT females mated with WT or *SPSL1*-mutant males (Fig 4A–4D’). In females mated with WT males, the spermatophore had a balloon-like structure composed of a swollen corpus and a tube-like collum (Fig 4C), similar to that in lepidopteran species previously reported [22, 31]. However, to our surprise, in females mated with *SPSL1*-mutant males, there were severe defects in spermatophore formation. The membranes of spermatophores in females mated with *SPSL1*-mutant males were fragile and incomplete, and sperm were observed outside the spermatophores (Fig 4C’ and 4G).

The spermatophore in lepidopteran insects is a reaction vessel for sperm activation [20, 32, 33]. The activation of sperm results in vigorous motility of apyrene sperm and dissociation of eupyrene sperm bundles in the spermatophore [27, 34]. We then investigated whether *SPSL1* mutation affects sperm activation in spermatophore and found that, in WT females mated with *SPSL1*-mutant males, eupyrene sperm bundles failed to dissociate (Fig 4E), and most apyrene sperm were motionless compared to those mated with WT males (S1 and S2 Movies). We then quantified the frequencies of undissociated, dissociating, and dissociated eupyrene sperm bundles and found that the frequencies of dissociating and dissociated eupyrene sperm bundles were reduced in females mated with *SPSL1*-mutant males compared to those mated with WT males (Fig 4F). We also analyzed sperm activation in spermatophore-excluded copulatory bursae samples and found that the frequencies of undissociated and dissociated eupyrene sperm bundles were increased in those from females mated with *SPSL1*-mutant males compared to those mated with WT males (Fig 4G and 4H). Moreover, the number of sperm was counted in the copulatory bursae of females mated with WT or *SPSL1*-mutant males (Fig 4I–4K), and a significant decrease of the number of dissociated eupyrene sperm was detected in the copulatory bursae of females mated with *SPSL1*-mutant males (Fig 4K). These results showed that *SPSL1* is involved in spermatophore formation and sperm activation in the female reproductive system.

### *SPSL1* is involved in free amino acid and urea regulation in copulatory bursa

**Fig 5:**
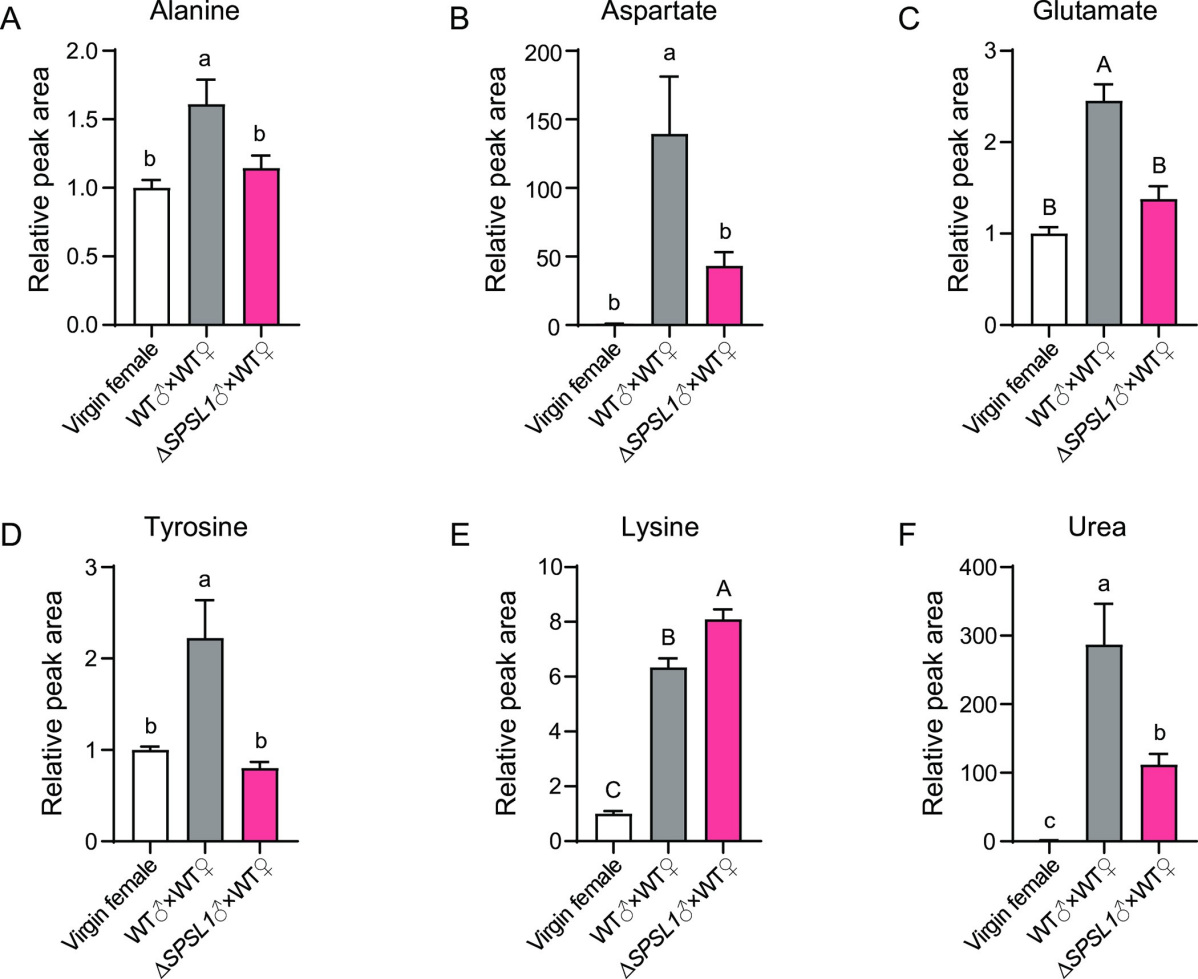
Analysis of free amino acids and urea using LC-MS/MS. (A-F) The relative peak areas of A) alanine, B) aspartate, C) glutamate, D) tyrosine, E) lysine, and F) urea detected in the copulatory bursae of virgin females, WT females mated with WT males, and WT females mated with *SPSL1*-mutant males. Five biological replicates were performed. Data were normalized to the relative peak area of the virgin females. Data represent means ± SEM. Uppercase letters, *p* < 0.01; Lowercase letters, *p* < 0.05. One-way ANOVA test. Δ*SPSL1* represents *SPSL1* mutants.

During sperm maturation in the lepidopteran model insect *Bombyx mori*, accumulation of alanine, urea, succinate, and other metabolites result from proteolysis reactions, may provide an energy source for sperm activation [20, 33, 35]. To gain insights into whether *SPSL1* is involved in proteolysis reactions in the copulatory bursae, we analyzed key small-molecule metabolites using liquid chromatography with tandem mass spectrometry (LC-MS/MS). Of the 18 metabolites detected, lysine, alanine, aspartate, glutamate, tyrosine, and urea were detected at significantly different levels between WT females that had been mated with WT compared to *SPSL1*-mutant males (Figs 5 and S3). With exception of lysine, these metabolites were slightly induced after mating with *SPSL1*-mutant males but were dramatically enriched after mating with WT males (Fig 5A–5F). These results suggest that SPSL1 functions in proteolysis reactions that regulate sperm activation, and the defects in sperm motility and dissociation observed in female copulatory bursae after mating with *SPSL1*-mutant males are likely due to the reduced amounts of free amino acids and urea that are required for sperm maturation.

### *SPSL1* mutation leads to failure in sperm migration into the spermatheca

**Fig 6:**
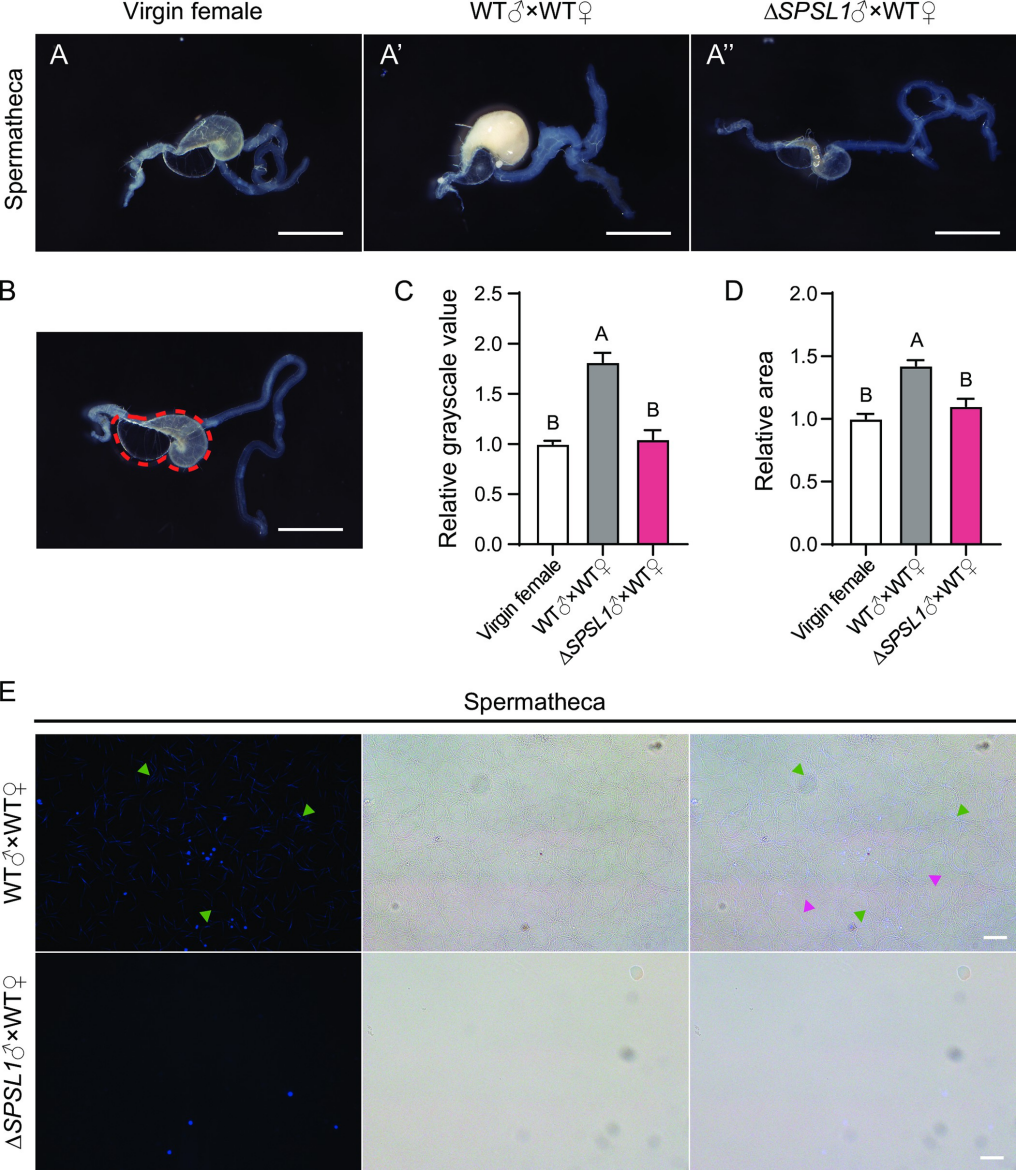
Mutation of *SPSL1* induces failure of sperm transfer into the spermatheca. (A-A”) Representative images of spermatheca of A) virgin females, A’) WT females mated with WT males, and A”) WT females mated with *SPSL1*-mutant males. Scale bars, 1mm. (B) Representative image of spermatheca indicating area used for quantification within red dotted lines. Scale bar, 1mm. (C) The relative grayscale value of spermathecae of virgin females and females for indicated matings. (D) The relative area of spermathecae of virgin females and females for indicated matings. n > 15. Different Uppercase letters, *p* < 0.01. One-way ANOVA test. (E) Representative images of sperm released from the spermatheca at 4 hpc. Green arrows, eupyrene sperm; Magenta arrows, apyrene sperm. Blue, Hoechst. Scale bars, 20 μm. Δ*SPSL1* represents *SPSL1* mutants.

After sperm activation, the apyrene sperm and the dissociated eupyrene sperm move from the copulatory bursa to the spermatheca for storage and fertilization [36, 37]. We next investigated whether *SPSL1* mutation affects sperm transfer into spermatheca. We found that at 4 hpc the spermathecae of females mated with WT males contained sperm, whereas the spermathecae of virgin females and females mated with *SPSL1*-mutant males were empty (Fig 6A–6A”). Quantitative analyses showed that the relative grayscale value and the relative area of the spermathecae of females mated with *SPSL1*-mutant males were significantly lower than those in females mated with WT males; levels in the former were comparable to those of virgin females (Fig 6B–6D). We also analyzed the outflow from the spermathecae at 4 hpc after fluorescence staining. We observed intertwined eupyrene sperm and apyrene sperm in the spermathecae of females mated with WT males but not in the spermathecae of females mated with *SPSL1*-mutant males (Fig 6E). To exclude the possibility of delayed transfer of sperm into the spermathecae, the outflow from the spermathecae was analyzed at 24 hpc, and no sperm was detected in the spermathecae of females mated with *SPSL1*-mutant males (S4 Fig). These data imply that sperm fail to transfer into spermatheca in the absence of SPSL1.

## Discussion

**Fig 7:**
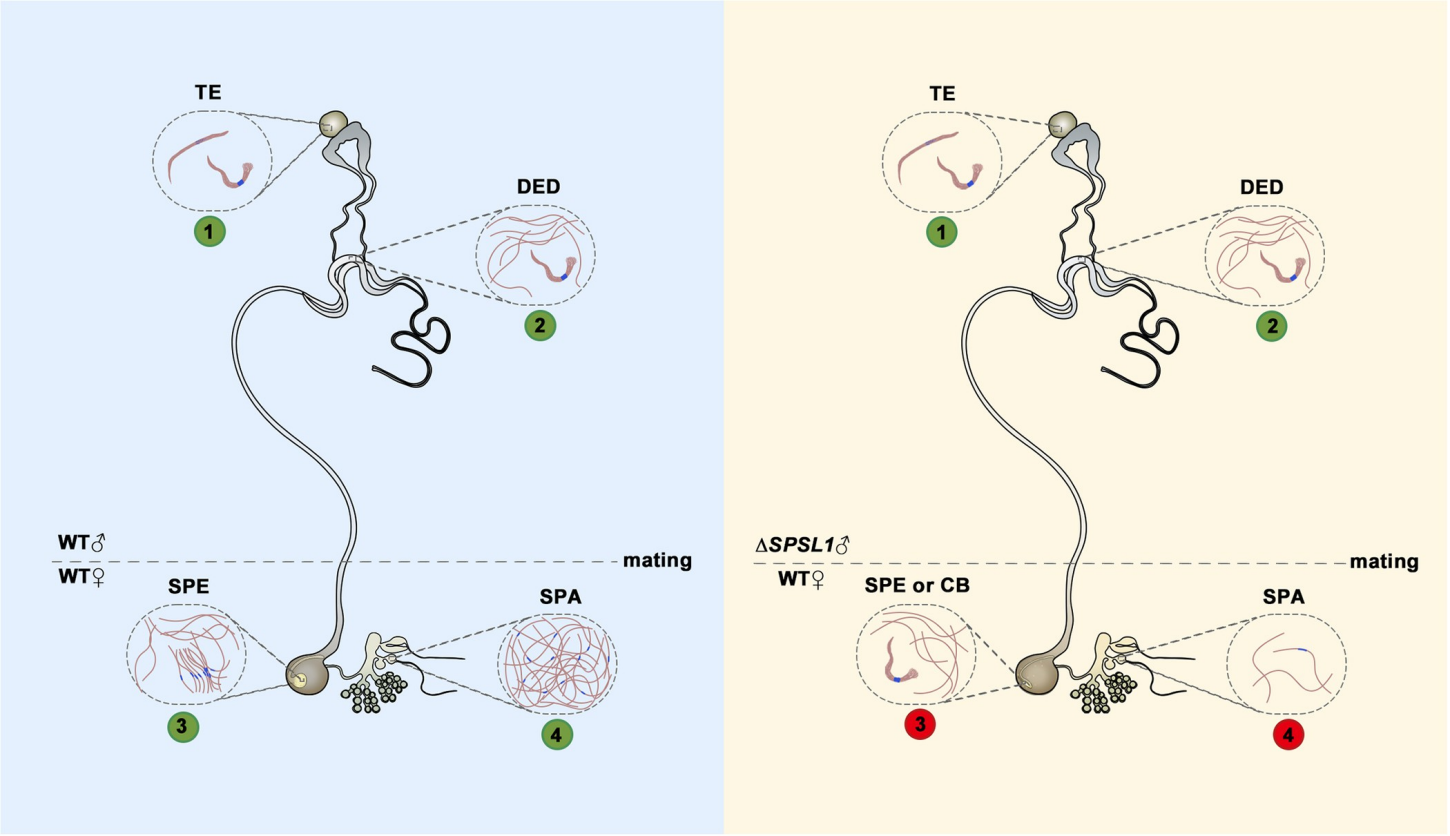
Model illustrating SPSL1-mediated regulation of male fertility in *S*. *frugiperda*. Spermatogenesis and sperm migration are normal in the reproductive system of WT or *SPSL1*-mutant males (green dot 1 and 2). In the copulatory bursa of females mated with WT males, the spermatophore is formed, and eupyrene sperm bundles are dissociated in the spermatophore during copulation (green dot 3). Apyrene sperm and dissociated eupyrene sperm are then transferred to the spermatheca for fertilization (green dot 4). In the copulatory bursa of females mated with *SPSL1*-mutant males, the spermatophore is malformed, and eupyrene sperm bundles fail to dissociate during copulation (red dot 3); thus, few sperm are transferred to the spermatheca for fertilization (red dot 4). Abbreviations: TE, testis; DED, double ejaculation ductus; SPE, spermatophore; CB, copulatory bursa; SPA, spermatheca. Δ*SPSL1* represents *SPSL1* mutants.

Trypsin-type serine proteases are known as components transferred from males to females and are crucial for fertility in diverse species [24–26, 38]. Despite our previous work found that the mutation of *Ser1* causes male sterility in *B*. *mori* [28], how its particular role in the reproductive process of any lepidopteran pest remains incompletely known. The effects on the reproductive tissues and cells induced by the mutation, as well as the biochemical reactions it may involve in are not investigated. In this study, we demonstrated that *SPSL1* is involved in the regulation of spermatophore formation and sperm activation in *S*. *frugiperda* females. We generated loss-of-function mutants of *SPSL1* via CRISPR/Cas9-based genome editing. No gross morphological or developmental defects were observed in the mutant females or males, and fluorescence staining of dichotomous sperm bundles revealed no obvious defects in *SPSL1*-mutant males. However, fecundity was dramatically reduced in females mated with *SPSL1*-mutant males, and our analyses indicated that the formation of the spermatophore was disrupted and that activation of both eupyrene sperm and apyrene sperm was severely inhibited. Our mass spectrometry analysis showed that levels of energy-related metabolites were significantly reduced in the copulatory bursae of females mated with *SPSL1*-mutant males. Furthermore, eupyrene sperm and apyrene sperm were barely detected in the spermathecae of females mated with *SPSL1*-mutant males, indicating that sperm migration was impeded. We therefore propose that SPSL1 is an integral component for spermatophore formation and sperm activation during copulation in *S*. *frugiperda* (Fig 7). Taken together, our study reveals a molecular mechanism in the regulation of spermatophore formation in lepidopteran insects, and gives a panoramic view of how sperm migration has been affected by SPSL1 from male reproductive system to female reproductive system.

How SPSL1 participates in spermatophore formation and sperm activation? Spermatophore is a unique structure in the reproductive system of lepidopterans, and the physiological and biochemical reactions that occur in the spermatophore during copulation are mostly uncharacterized [22, 31]. To our knowledge, SPSL1 is the first SFP identified that is involved in the formation of the spermatophore. We found that lack of SPSL1 leads to incomplete formation of the membrane of the spermatophore during copulation, and therefore sperm were observed outside the spermatophore. The spermatophore is reported as a structure where sperm activation takes place [20], however, our results lead us to ask whether the formation of spermatophore depends on sperm activity. Apyrene sperm have been shown to play a role in the dissociation of eupyrene sperm bundles [17, 18], and we observed that apyrene sperm motility was reduced in the females mated with *SPSL1*-mutant males. Since alanine and urea are products of the arginine degradation cascade that occurs in the spermatophore [20], we hypothesize that SPSL1 hydrolysates may provide energy for apyrene sperm motility, which promotes eupyrene sperm dissociation and spermatophore formation. It is also possible that SPSL1 or its hydrolysates may act as spermatophore structural proteins and that the failure of spermatophore formation leads to inactivated sperm. These possibilities should be explored in further studies.

In *Drosophila*, Sex Peptide (also known as Acp70) is a SFP that regulates the post-mating behavior of females including sexual acceptance and oviposition [5, 6]. Genetic and biochemical evidence indicated that the function of Sex Peptide in female *Drosophila* depends on a processing by a trypsin-mediated cascade [39]. In our study, we observed that the number of eggs laid by females mated with *SPSL1*-mutant males was significantly lower than the number laid by females mated with WT males. This may be due to decreased migration of sperm or sperm-localized SFPs into spermatheca and therefore failure to stimulate post-mating behavior in females. It is possible that SPSL1 is involved in the regulation of a putative Sex Peptide in *S*. *frugiperda* and lack of SPSL1 deactivates the Sex Peptide and leads to defects in oviposition. Efforts to identify substrates of SPSL1 and exploration of their functions will be the focus of further studies.

## Materials and methods

### *Spodoptera frugiperda* strains and rearing

The *S*. *frugiperda* strains were obtained from Prof. Shutang Zhou (Henan University, College of Life Science) and reared in the laboratory on an artificial diet under conditions of 25 ± 1°C, 60 ± 10% relative humidity, and a 14 h light:10 h dark photoperiod. Moths were fed with 10% sucrose solution after eclosion.

### Phylogenetic and amino acid alignment analysis

The phylogenetic tree of the SPSL1 proteins was generated using the neighbor-joining algorithm in MEGA X with a bootstrap of 1000 replications [40]. The evolutionary distances were computed using the Poisson correction method and are in units of the number of amino acid substitutions per site. The sequences were aligned using the ClustalW and the GENEDOC [41]. The protein sequences used to create the diagram were downloaded from National Center for Biotechnology Information and were listed in S1 Table.

### RNA isolation, cDNA synthesis, and qRT-PCR

Total RNA was extracted from the reproductive system of the male adults using Trizol Reagent (Invitrogen, USA) according to the manufacturer’s instructions. The cDNAs were synthesized using PrimeScript RT reagent kit (TaKaRa, China) in a 20 μL reaction mixture containing 1 μg of total RNA.

Quantitative real-time RT-PCR (qRT-PCR) was performed using SYBR Green Realtime PCR Master Mix (Thermo Fisher Scientific, USA) on a StepOnePlus Real-Time PCR system (Applied Biosystems, USA). The quantitative variations were evaluated by the relative quantification method (2^-ΔΔCt^). Sequences of the qRT-PCR primers are listed in S2 Table.

### CRISPR/Cas9 genome editing and mutation detection

Two 23-nucleotide single-guide RNAs (sgRNA) targeting *SPSL1* were designed according to the GGN19GG rule. The sgRNAs and Cas9 mRNA were synthesized and purified as described [42]. Newly laid eggs were microinjected with 300 ng/μL of Cas9 mRNA and 300 ng/μL of each sgRNA within 3 h of oviposition. About 1.6–2.0 ng of total RNA was injected into each egg. The eggs were incubated at 25°C.

Genomic DNA extracted from injected eggs (n = 30) at 48 h after injection was used as a template to amplify the sgRNA target sites with specific primers (S2 Table). A roughly 800 bp fragment flanking the target sites was amplified by PCR, and the products were directly sequenced or ligated into a pMD-18T vector for sequencing. The mutation of each insect used for experiment was verified.

### Fertility assay

Males on the second day after eclosion were paired with females on the third day after eclosion. Each pair of insects was kept in a plastic bag (25 cm × 35 cm) and fed with 10% sucrose solution. Eggs laid by successfully mated females were manually counted at 48 h-intervals until the death of the female. The hatching rate was calculated by using the number of eggs laid and hatched. The experiment was performed in triplicate, and each replicate used at least 10 pairs of moths.

### Fluorescence staining

Sperm bundles were isolated from the testes of male adults on the second day after eclosion. The collected sperm bundles were fixed with immunol staining fix solution (Beyotime, China) for 1 h. After two washes with PBS, samples were incubated in blocking solution (1 × PBS + 0.1% Triton X-100 + 0.5% bovine serum albumin) for 1 h. After a wash with PBS, samples were incubated with TRITC Phalloidin (YEASEN, China) for 1 h, and then with Hoechst (Beyotime, China) for 20 min at room temperature. The samples were further washed two times with PBS and subsequently mounted in antifade medium (YEASEN, China). Sperm isolated from double ejaculation ductus of virgin males (second day after eclosion), and copulatory bursae, spermatophores, and spermathecae of 1 hpc, 1 hpc, and 4 hpc females, respectively, were smeared on a micro-slide and observed by microscopy after Hoechst (Beyotime, China) staining. Images were taken with a Nikon (Japan) Ti-E or an Olympus (Japan) BX53 fluorescence microscope.

### Analysis of sperm activation

Apyrene sperm were collected in the spermatophores 1 hpc and imaged using a Nikon Ti-E fluorescence microscope. Eupyrene sperm were collected from spermatophores or spermatophore-excluded copulatory bursae of 1 hpc females and smeared on a square area (1.5 cm × 1.5 cm) of a micro-slide. The samples were imaged using a Nikon Ti-E fluorescence microscopy in 20× fields of view using the five-point sampling method. The occurrence frequency and amount of eupyrene sperm bundles, dissociating eupyrene sperm bundles, and dissociated eupyrene sperm were calculated by analysis of at least 30 fields of 20x view.

### LC-MS/MS analysis

The copulatory bursae were dissected from virgin females or mated females 1 hpc on the third day after eclosion and stored at -80°C. Samples were dried at -40°C for over 8 h in a lyophilizer (Labconco, USA). One sample was about 10 mg, and 5 samples from each condition were analyzed. Each sample was grounded in 300 μL 80% methanol (Sigma-Aldrich, USA) and treated with ultrasound at 0°C for 10 min. After centrifugation at 16800 g at 4°C for 5 min, 100 μL of the supernatant was collected and added with 50 μL acetonitrile (Sigma-Aldrich, USA) to precipitate overnight at -80°C. After centrifugation at 16800 g for 10 min, 100 μL of the supernatant was collected for analysis by LC-MS/MS. The analysis was performed on a UPLC instrument combined with a QTRAP 6500^+^ MS system (AB SCIEX, USA) equipped with an electrospray ionization (ESI) source (AB SCIEX, USA). Instrument control and data acquisition were performed using Analyst 1.6.3 software (AB SCIEX, USA), and data processing was performed using MultiQuant 3.0.2 software (AB SCIEX, USA). The amino acids and urea were separated on an ACQUITY UPLC BEH Amide column (1.7 μm, 2.1 x 100 mm, Waters, USA). Free amino acid mixed standard (AAS18, Sigma Aldrich, USA) and urea standard (U5128, Sigma Aldrich, USA) were used for qualitative analysis. The UPLC methods for different amino acids and urea are shown in S3 Table. The optimized ESI operating parameters for positive mode were: IS, 5500 V; CUR, 35 psi; TEM, 500°C; GS1, 55 psi; GS2, 55 psi. All analytes were detected using multiple reaction monitoring (MRM) mode, and the specific MRM parameters for each analyte are given in S4 Table.

### Data analysis

Data were analyzed using GraphPad Prism, version 9. All data are represented as the mean ± standard error (SEM). Statistically significant differences were represented by asterisks as *p < 0.05, **p < 0.01, ***p < 0.001.

## Supporting information

S1 Fig Phylogenetic analysis and sequence alignment of SPSL1. (A) Phylogenetic tree of SPSL1 based on the alignment of amino acid sequences of the nineteen insect species using the neighbor-joining algorithm with a bootstrap of 1000 replications. Names and accessions used in the tree are listed in S1 Table. (B) Multiple alignment of the putative SPSL1 protein sequences from the seven lepidopteran insects. The highly conserved regions are highlighted by capital letters.
(TIF)

S2 Fig *SPSL1*-mutant male does not have gross defects in the reproductive system. (A, A’) Representative images of the reproductive system of WT and *SPSL1*-mutant males on the second day after eclosion. Scale bars, 1 cm. (B, B’) Representative images of the single ejaculation ductus (SED) of WT and *SPSL1*-mutant males on the second day after eclosion. Scale bars, 0.5 cm. Δ*SPSL1* represents *SPSL1* mutants.
(TIF)

S3 Fig Analysis of free amino acids using LC-MS/MS. (A-L) The relative peak areas of A) cystine, B) histidine, C) isoleucine, D) leucine, E) proline, F) serine, G) valine, H) arginine, I) glycine, J) methionine, K) phenylalanine, and L) threonine detected in the copulatory bursae of virgin WT females, WT females mated with WT males, and WT females mated with *SPSL1*-mutant males. Data were normalized to the relative peak area of the virgin females. Five biological replicates were performed. Data represent means ± SEM; Uppercase letters, *p* < 0.01; Lowercase letters, *p* < 0.05. One-way ANOVA test. Δ*SPSL1* represents *SPSL1* mutants.
(TIF)

S4 Fig Mutation of *SPSL1* leads to failure of sperm transfer into spermatheca at 24 hpc. Representative images of sperm released from the spermatheca at 24 hpc. Green arrows, eupyrene sperm; Magenta arrows, apyrene sperm. Blue, Hoechst. Scale bars, 50 μm. Δ*SPSL1* represents *SPSL1* mutants.
(TIF)

S1 Table List of species and accession number. (XLSX)

S2 Table Primers used in this study. (XLSX)

S3 Table The UPLC methods for different amino acids and urea. A: 95% water, 5% acetonitrile, 10 mM ammonium formate, 0.1% formic acid. B: 95% acetonitrile, 5% water, 10 mM ammonium formate, 0.1% formic acid. The column oven was set at 40°C.
(XLSX)

S4 Table The specific MRM parameters for each analyte. Q1 is parent ion, Q3 is the daughter ion, CE is the collision energy, DP is the declustering potential. For each compound, the Q1 and the first Q3 were used for quantification, the second as a quality control.
(XLSX)

S1 Movie Behavior of apyrene sperm in the spermatophores 1 hpc of WT females mated with WT males. Scale bar, 50 μm.
(MP4)

S2 Movie Behavior of apyrene sperm in the spermatophores 1 hpc of WT females mated with *SPSL1* mutant males. Scale bar, 50 μm.
(MP4)
