# Supplementary figures and images for: SPSL1 is essential for spermatophore formation and sperm activation in Spodoptera frugiperda
Source: PLoS Genet. 2023 Dec 4;19(12):e1011073. doi: 10.1371/journal.pgen.1011073 (PMC10721193; doi:10.1371/journal.pgen.1011073)

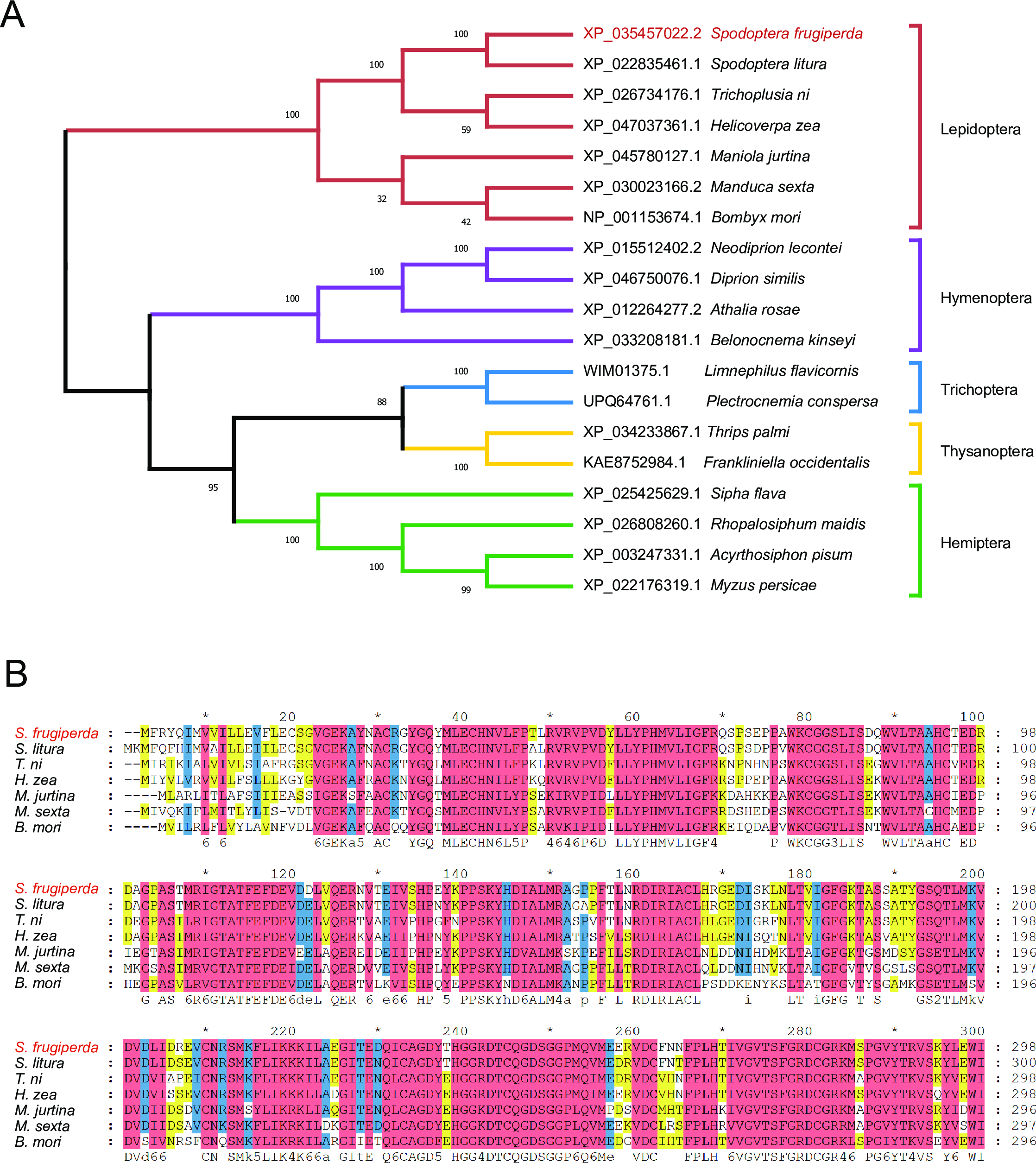

Supplement: S1 Fig — (A) Phylogenetic tree of SPSL1 based on the alignment of amino acid sequences of the nineteen insect species using the neighbor-joining algorithm with a bootstrap of 1000 replications. Names and accessions used in the tree are listed in S1 Table. (B) Multiple alignment of the putative SPSL1 protein sequences from the seven lepidopteran insects. The highly conserved regions are highlighted by capital letters. (TIF) [file pgen.1011073.s001.tif]

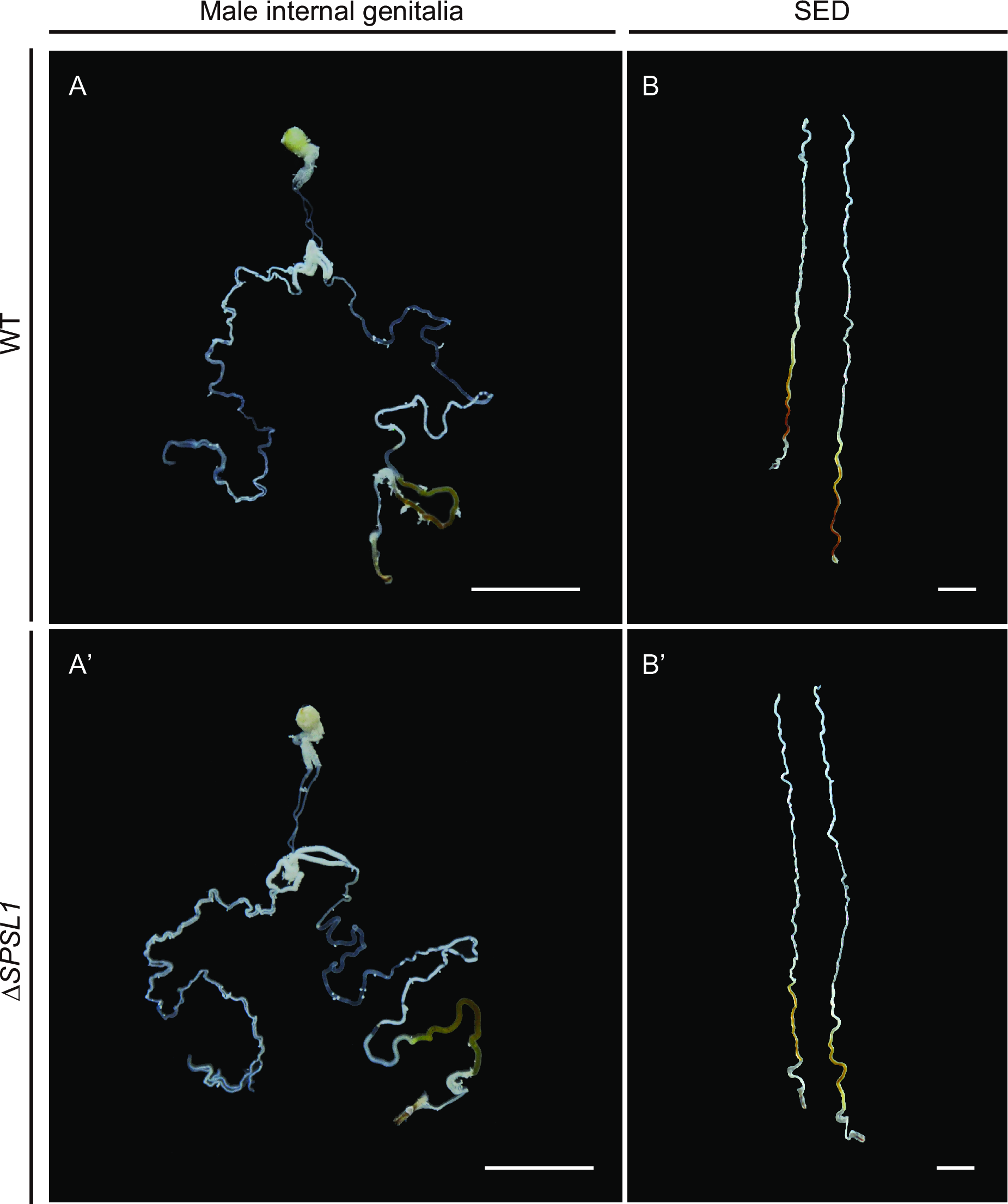

Supplement: S2 Fig — (A, A’) Representative images of the reproductive system of WT and SPSL1-mutant males on the second day after eclosion. Scale bars, 1 cm. (B, B’) Representative images of the single ejaculation ductus (SED) of WT and SPSL1-mutant males on the second day after eclosion. Scale bars, 0.5 cm. ΔSPSL1 represents SPSL1 mutants. (TIF) [file pgen.1011073.s002.tif]

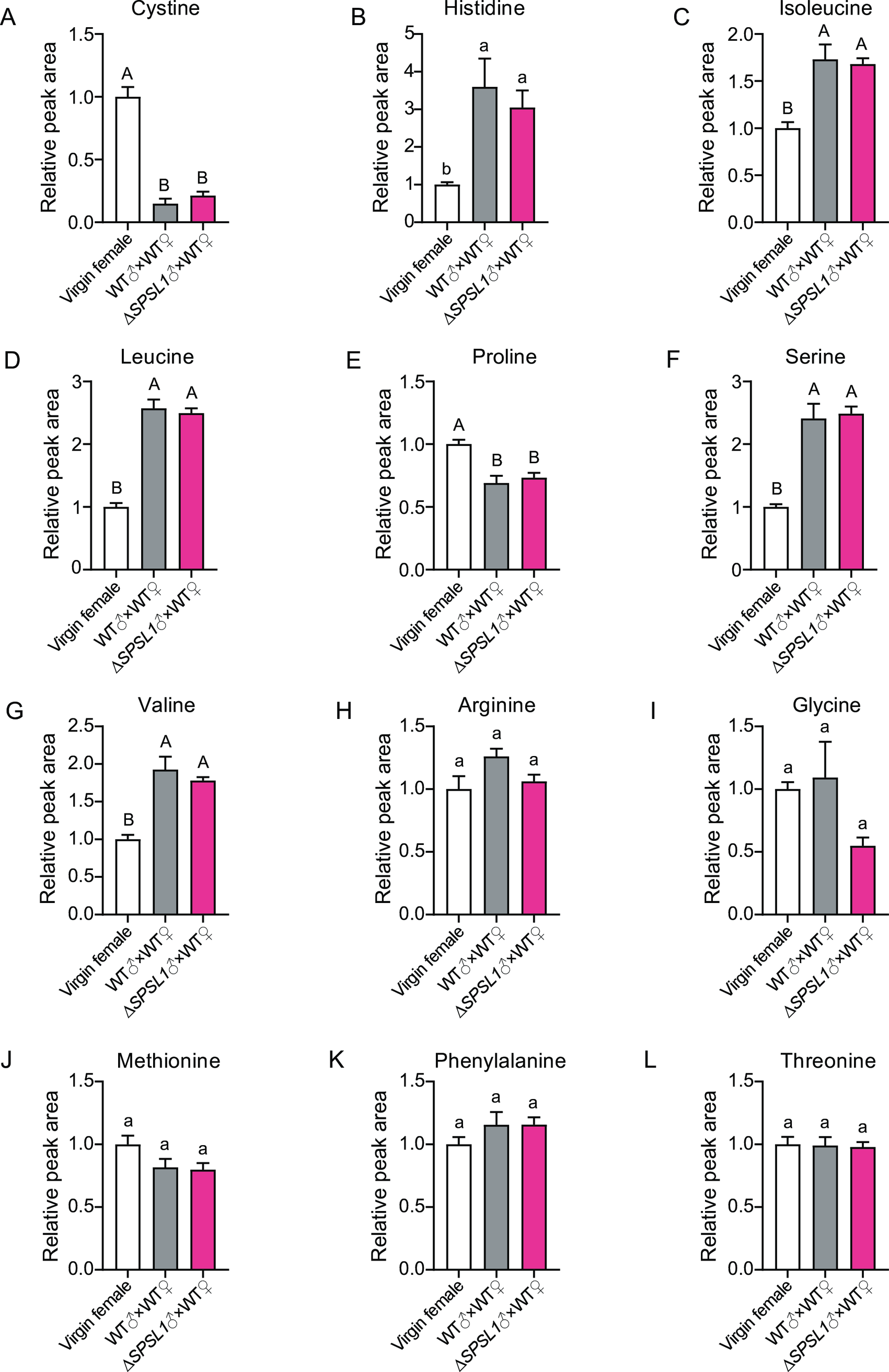

Supplement: S3 Fig — (A-L) The relative peak areas of A) cystine, B) histidine, C) isoleucine, D) leucine, E) proline, F) serine, G) valine, H) arginine, I) glycine, J) methionine, K) phenylalanine, and L) threonine detected in the copulatory bursae of virgin WT females, WT females mated with WT males, and WT females mated with SPSL1-mutant males. Data were normalized to the relative peak area of the virgin females. Five biological replicates were performed. Data represent means ± SEM; Uppercase letters, p < 0.01; Lowercase letters, p < 0.05. One-way ANOVA test. ΔSPSL1 represents SPSL1 mutants. (TIF) [file pgen.1011073.s003.tif]

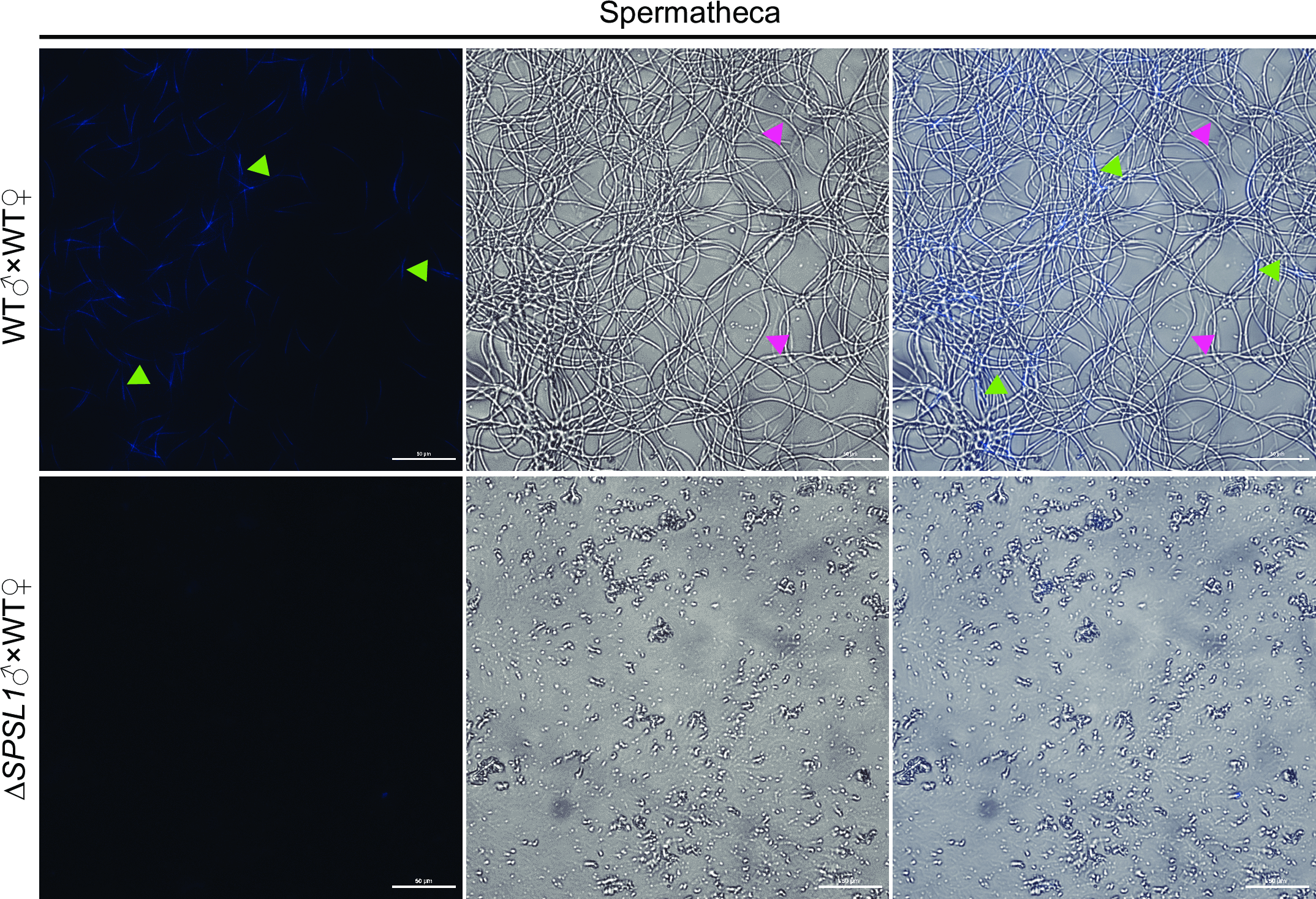

Supplement: S4 Fig — Representative images of sperm released from the spermatheca at 24 hpc. Green arrows, eupyrene sperm; Magenta arrows, apyrene sperm. Blue, Hoechst. Scale bars, 50 μm. ΔSPSL1 represents SPSL1 mutants. (TIF) [file pgen.1011073.s004.tif]
